# Vaginal microbicides: detecting toxicities *in vivo *that paradoxically increase pathogen transmission

**DOI:** 10.1186/1471-2334-6-90

**Published:** 2006-06-01

**Authors:** Richard A Cone, Timothy Hoen, XiXi Wong, Raed Abusuwwa, Deborah J Anderson, Thomas R Moench

**Affiliations:** 1 Mucosal Protection Laboratory, Dept. of Biophysics, Johns Hopkins University, Baltimore, MD 21218, USA; 2 ReProtect, Inc., Baltimore, MD 21286, USA; 3 Obstetrics and Gynecology, Boston University Medical Campus, Boston, MA 02118, USA

## Abstract

**Background:**

Microbicides must protect against STD pathogens without causing unacceptable toxic effects. Microbicides based on nonoxynol-9 (N9) and other detergents disrupt sperm, HSV and HIV membranes, and these agents are effective contraceptives. But paradoxically N9 fails to protect women against HIV and other STD pathogens, most likely because it causes toxic effects that increase susceptibility. The mouse HSV-2 vaginal transmission model reported here: (a) Directly tests for toxic effects that increase susceptibility to HSV-2, (b) Determines *in vivo *whether a microbicide can protect against HSV-2 transmission *without *causing toxicities that increase susceptibility, and (c) Identifies those toxic effects that best correlate with the increased HSV susceptibility.

**Methods:**

Susceptibility was evaluated in progestin-treated mice by delivering a low-dose viral inoculum (0.1 ID_50_) at various times after delivering the candidate microbicide to detect whether the candidate *increased *the fraction of mice infected. Ten agents were tested – five detergents: nonionic (N9), cationic (benzalkonium chloride, BZK), anionic (sodium dodecylsulfate, SDS), the pair of detergents in C31G (C_14_AO and C_16_B); one surface active agent (chlorhexidine); two non-detergents (BufferGel^®^, and sulfonated polystyrene, SPS); and HEC placebo gel (hydroxyethylcellulose). Toxic effects were evaluated by histology, uptake of a 'dead cell' dye, colposcopy, enumeration of vaginal macrophages, and measurement of inflammatory cytokines.

**Results:**

A single dose of N9 protected against HSV-2 for a few minutes but then rapidly increased susceptibility, which reached maximum at 12 hours. When applied at the minimal concentration needed for brief partial protection, all five detergents caused a subsequent increase in susceptibility at 12 hours of ~20–30-fold. Surprisingly, colposcopy failed to detect visible signs of the N9 toxic effect that increased susceptibility at 12 hours. Toxic effects that occurred contemporaneously with increased susceptibility were rapid exfoliation and re-growth of epithelial cell layers, entry of macrophages into the vaginal lumen, and release of one or more inflammatory cytokines (Il-1β, KC, MIP 1α, RANTES). The non-detergent microbicides and HEC placebo caused no significant increase in susceptibility or toxic effects.

**Conclusion:**

This mouse HSV-2 model provides a sensitive method to detect microbicide-induced toxicities that increase susceptibility to infection. In this model, there was no concentration at which detergents provided protection without significantly increasing susceptibility.

## Background

The healthy, intact vaginal epithelium provides a significant barrier against infection: To transmit infection reliably, 10,000 times more SIV must be delivered to the macaque vagina than that required intravenously [1], and in humans, heterosexual transmission of HIV is estimated to occur in only ~1/1000 coital acts [2]. Even when the male partner is in the highly infectious acute phase, transmission is estimated to occur in only ~1/50 coital acts [3]. Thus any microbicide that has a toxic effect that compromises the vaginal barrier can cause a major increase in susceptibility. To help identify promising microbicide candidates for future efficacy trials, preclinical screening tests are needed that will disclose any toxic effect that increases vaginal susceptibility to infection. At present, screening for toxic effects *in vivo *is most often performed in the primate, rabbit, and mouse vaginal irritation models [4-9]. These models employ histology, colposcopy, and the release of inflammatory cytokines to detect toxic effects [4-6,8-12]. Although of obvious importance, these toxic effects serve only as surrogates for susceptibility, and thus they may fail to disclose toxic effects that significantly increase susceptibility to infection. Fortunately, animal models can be devised to test directly whether a microbicide increases susceptibility.

Some readily observed toxic effects are very likely to increase susceptibility, such as ulceration and inflammatory conditions that increase access to target cells, but there may also be additional susceptibility-increasing toxic effects that have not yet been identified or observed; for example, toxic effects that increase the density of cell-surface receptors on target cells, or that increase the amplification of nascent infections. With this in mind, we developed a mouse vaginal model to detect whether candidate microbicides cause, *by any mechanism*, toxicity that increases the susceptibility of the vagina to HSV infections. HSV-2, like HIV, is an enveloped virus that can transmit infections through the epithelium, and genital herpes is a major STI that is a major co-factor for HIV transmission [13]. HSV-2 is a human pathogen that readily infects many other species, including mice, by binding to highly conserved entry receptors, especially nectin-1, on epithelial target cell surfaces. For example, human, bovine, and porcine herpes viruses can all use human, mouse and porcine forms of nectin-1 for cell entry [14]. Moreover, the course of infection is similar in mice and humans. These characteristics strongly suggest that candidate microbicides that increase HSV susceptibility in mice will likely increase HSV susceptibility in humans.

HIV and HSV both bind initially to heparan sulphate-like molecules on the surfaces of their target cells [15], and although these viruses then use different entry receptors, toxic effects that increase the presence of, or access to, heparan sulphate receptors on their target cells might possibly increase susceptibility to both viruses. Similarly, sulfonated and sulfated polysaccharides (PRO 2000, SPS, cellulose sulphate, carrageenan), bind to both viruses, and PRO 2000 protects against both HIV [16] and HSV [17] in animal models and against both HIV and HSV in human ex vivo tests [18]. Thus toxic effects that increase HSV susceptibility in the mouse may in some cases also reveal toxic effects that increase HIV susceptibility [19].

A key paper by Phillips and Zacharopoulus [20] on susceptibility to rectal transmission of HSV in the mouse revealed that nonoxynol-9 (N9) not only failed to protect, but caused rapid exfoliation of sheets of epithelial cells and greatly increased rectal susceptibility. Phillips and colleagues then found [21,22] that N9 causes similar rapid exfoliation of rectal epithelium in humans. Their results strongly suggest that N9 should not be used for rectal protection in humans. In testing microbicides for protective efficacy in a mouse chlamydia model [23], we found that chlorhexidine (CHX), a widely used microbicidal preservative, causes a 100-fold increase in vaginal susceptibility to Chlamydia trachomatis when tested three *days *after a single vaginal application of this surface-active agent. Thus mouse models for vaginal transmission of HSV and chlamydia provide a way to detect *in vivo *toxicity that increases vaginal susceptibility to viral as well as bacterial STD pathogens. The protective efficacy of many candidate microbicides has been tested for HSV and Chlamydia in a variety of mouse models [7,24-28]. The present investigation is the first designed to: (a) Detect susceptibility-increasing toxicities by observing susceptibility as a function of time following a single application of candidate microbicides, (b) Test whether there is a dose range of a candidate microbicide that can prevent vaginal HSV transmission without causing toxicities that increase susceptibility to HSV, and (c) Monitor observable toxic effects that could potentially be used in clinical (Phase I) evaluations of candidate microbicides to identify toxic effects that best correlate with toxicities that increase vaginal susceptibility to HSV.

## Methods

### Microbicide candidates

A cross-section of candidate microbicides was selected, some of which have been reported to protect against HSV-2 in mouse vaginal transmission models: N9 [28], SPS [26] (which is similar to T-PSS [24]), chlorhexidine [26], and BufferGel [29]. Others were selected based on published *in vitro *tests: C31G inactivates HSV-2 [30] and both detergents in C31G, (C_14_AO+C_16_B), inactivate HIV [4,31]; BZK and SDS both inactivate HIV [10,31]. Several detergents were selected since detergents, especially N9, are the candidates for which the most clinical data are available. All five of the detergents are comparably potent at inactivating HSV, HIV, or sperm *in vitro*: ~0.01–0.03% rapidly disrupts envelope and cell membranes [4,32-34]. Nonoxynol-9 is a non-ionic detergent used in most currently available vaginal spermicides and detergent-coated condoms, and is widely used in disinfectant handwashes. Benzalkonium Chloride (BZK) is a cationic detergent with exceptionally broad microbicidal action and is a component of the "Protectaid^®^" contraceptive sponge. Sodium Dodecyl Sulfate (SDS, also called sodium lauryl sulfate, SLS) is an anionic detergent widely used in toothpastes, shampoos and shaving creams. C31G is an equimolar mixture of two detergents (C_14_AO, C14 alkyl amine oxide and C_16_B, C16 alkyl betaine) used as an oral disinfectant in dentistry and as the active ingredient in "Savvy", a spermicidal microbicide now in clinical efficacy trials for contraception and HIV prevention. Chlorhexidine (CHX) is a surface active agent that is even more potent than the above detergents and is used as a preservative in K-Y Jelly^® ^and Surgilube^®^, and as a disinfectant in mouthwashes and antimicrobial handwashes. BufferGel^®^ is a spermicidal microbicidal gel made with Carbopol 974P, a cross-linked polyacrylic acid polymer, and formulated at pH 3.9 to match the pH of the healthy human vagina. It has adequate buffer capacity to acidify the ejaculate and thereby reinforces the protective acidity of the vagina. Sulfonated polystyrene (SPS) is a polymer similar to T-PSS [24], and has potent spermicidal and antiviral efficacy in vitro [26]. It is similar, to some extent, to the sulfated and sulfonated polysaccharides now in clinical HIV prevention trials (Carraguard, PRO 2000, and cellulose sulphate). These candidates were obtained from: N9 (Rhone-Poulenc, Cranbury NJ); BZK and chlorhexidine (Sigma, St. Louis, MO); SDS (Invitrogen Corp., Carlsbad, CA); C_14_AO (myristyl dimethylamine oxide, Chemron, Pasa Robles, CA.) and C_16_B (cetyldimethylbetaine, DeForest Enterprises, Inc., Boca Raton, FL); BufferGel^®^ and HEC placebo gel [35] (ReProtect, Inc., Baltimore MD); SPS (polystyrene sulfonate, average molecular weight 500,000: Scientific Polymer Products, Inc., Ontario, NY). With the exception of BufferGel and the HEC placebo gel, all agents were tested for toxicity as 2% solutions in phosphate buffered saline (PBS) to avoid the confounding effects of differing formulations. Similarly, for determining the acute toxicity and protective efficacy, the detergents and CHX were also delivered in PBS but at various concentrations as needed.

### Mouse model

#### Progestin treatment

Female CF-1 mice 6–8 weeks old (Harlan, Indianapolis, IN) were acclimatized for 1–2 weeks after shipping, then injected subcutaneously with 2.5 mg Depo-Provera^® ^(medroxyprogesterone acetate) (Pharmacia & Upjohn Company, Kalamazoo, MI.), a treatment that produces a diestrous-like state that eliminates the stratified squamous layer of dead and dying cells that otherwise helps protect the vagina. In this diestrous-like state the epithelium becomes similar to columnar epithelium in that the entire epithelial surface becomes covered with living cells. This progestin treatment greatly increases HSV susceptibility and makes mice more uniform in susceptibility than randomly cycling mice [26,28,36]. Depo-Provera makes the mouse vagina more closely mimic the most accessible HSV target cells in the human female genital tract, the columnar epithelial cells of the endocervical canal and regions of cervical ectopy that occur commonly in younger women, regions in which living cells are exposed directly on the face of the cervix.

#### Viral inoculum

Strain G of HSV-2 (ATCC lot #3405329) was obtained from Virotech International (Rockville, MD; 5 × 10^8 ^TCID_50_/ml). The viral stock was thawed and refrozen in 100 μl aliquots, then stored at -70°C. A thawed aliquot of viral stock was diluted with Bartels Tissue Culture Refeeding Medium (Trinity Biotech, St. Louis, MO) to yield an inoculum with 10 ID_50 _in a 10 μl inoculum (~10^4 ^TCID_50_). For low-dose inocula, the viral stock was further diluted with Bartels Medium as needed. The diluted viral stock was stored on ice and used within 1 hour of thawing. The 10 μl viral inoculum was delivered with a Wiretrol pipet (Drummond Scientific, Broomall, PA) with a fire-polished tip to minimize potential injury.

#### Assay for infection

As reported earlier [26,28,29], vaginal lavages were obtained 3 days after inoculation and evaluated for viral shedding. Input virus from the inoculum can not be detected for more than 6 hours after the inoculation, and viral shedding by infected animals reaches a maximum at 3 days (data not shown). This assay is more rapid and also more sensitive than waiting for visible lesions or death [26], both of which are more dependent on the hormonal and immune status of the mouse [37]. Importantly, assay of infection at day 3 avoids causing animal pain. Fifty μl of Bartels Medium was delivered to the vagina and pipetted in and out 20 times to maximize viral recovery, then diluted into 50 μl Bartels Medium in a 0.5 ml microfuge tube. The vaginal lavage samples were then spun at 6500 rpm for 5 minutes to pellet the cells and mucus. The pellet was then removed using a pipet tip to draw the pellet up the side of the tube and out of the supernatant. The supernatant was then placed on target cells (human newborn foreskin diploid fibroblast cells; Biowhitaker, Walkersville, MD). Cytopathic effect was scored 48 hours later, and mice whose lavage cultures displayed cytopathic effect were considered infected. The entire test thus requires a total of 5 days from inoculation to assay of infection, making this a relatively rapid and efficient animal model.

### Protection assay

Two procedures were used to observe the protective efficacy of the test agents. In the "mix externally" procedure, the 10 μl viral inoculum with 10 ID_50 _was mixed together with 20 μl of the test agent, incubated for 5 minutes at 37°C, and then this mixture was delivered to the vagina using a 50 μl Wiretrol pipet with fire polished tip. This method insures the virus is fully exposed to the test agent before it can contact target cells. In the "mix in vagina" procedure, which is intended to mimic human use of a microbicide, 20 μl of the test agent (or PBS) was delivered to the vagina and the 10 μl viral inoculum with 10 ID_50 _was delivered after a specified time interval. In most experiments 30 mice were divided into groups of 10, one group treated with one test agent, a second group with a second test agent, and the third (control) group was treated with PBS (phosphate buffered saline). Each such experiment was repeated two or three times to obtain results from 20 to 30 mice for each test agent.

### Susceptibility assay

In this procedure, 20 μl of the test agent was delivered to the vagina, and then a low-dose inoculum with 0.1 ID_50 _was delivered in 10 μl of Bartels medium after a specified time interval. To determine the relative susceptibility of the mice, two groups of control mice (treated with PBS at the same time interval) were used, one group inoculated with the same 0.1 ID_50 _low-dose inoculum and one inoculated with 10 ID_50_. On average, the low-dose inoculum infected 13.5% of the control mice; the high-dose inoculum infected 87%. For each test agent, the fraction infected in each of its control groups was used to construct a dose-response graph (fraction infected vs log ID) and drawing a linear interpolation between the fractions infected by the 0.1 and 10 ID_50 _inoculations. The fraction of mice infected in the test group was then plotted on this graph to determine the effective ID of the low-dose inoculum. Relative susceptibility is defined as the ratio of the effective ID the low-dose inoculum delivered to the test mice divided by the ID it delivered to control animals. For example, if the low-dose 0.1 ID_50 _inoculum infected 50% of the test mice, it acted effectively as 1 ID_50 _indicating the relative susceptibility of the test mice increased 10 fold. In most experiments 30 mice were divided into 3 groups of 8 (two test groups and 1 control group) all of which received the low-dose 0.1 ID_50 _inoculum, and one control group of 6 received the high-dose10 ID_50 _inoculum. Each experiment was repeated to obtain ~30–50 mice per group to insure robust statistical power.

### Acute toxicity

#### Dead cell dye

To detect acute toxicity to the epithelial cells, the vagina was stained with YOYO^®^-1 (Molecular Probes, Inc., Eugene, OR) a membrane impermeant dye that can enter the nuclei, and thereby become highly fluorescent, in cells whose membranes are disrupted. To keep the mice immobile throughout this test procedure, they were first anesthetized by intraperitoneal injection of 0.5 ml "Avertin" (2.5 g 2,2,2 tribromoethanol in 5 ml amylene hydrate and 200 ml water). Fifty μl of the test agent (or PBS control) was delivered with a Wiretrol pipet with fire-polished tip, and the agent left in place for 10 minutes. The vagina was then gently lavaged for 10 minutes using a syringe pump to continuously deliver PBS through a fire-polished Wiretrol pipet at a rate of 5 ml/minute. Then 20 μl of YOYO-1 (diluted to 5 μM with PBS) was delivered and left in place for 10 minutes before again lavaging the vagina for 10 minutes with PBS to remove unbound dye. The mouse was then sacrificed, the vagina dissected out and slit open lengthwise, and again rinsed for 10 minutes in PBS. The entire vagina was then mounted between two microscope slides that were squeezed together to flatten the vagina and open its numerous folds (rugae). Digital images of the flat-mounted vagina were taken with a macro-lens (1×) in a Nikon E800 epifluorescence microscope.

#### Histology

Groups of 4 mice were treated intra-vaginally with 20 μl 2% N-9 (or PBS for controls) and sacrificed at various times immediately before dissecting out the vagina. One N9 group and one control group were dissected at 10 minutes, and additional N9 groups were dissected 2, 8, 12, 18, and 24 hours post-exposure. Each vagina was fixed in 5 ml 10% neutral-buffered formalin (Sigma-Aldrich, St. Louis, MO). The vaginas were embedded, sectioned transversely at four regions, stained with hemotoxylin and eosin, examined by bright-field microscopy, and evaluated for average number of cell layers in the epithelium, and scored semi-quantitatively for quantity of visible cellular material in the lumen.

### Cytokine assay

The Bio-Plex system (Bio-Rad, Hercules, CA) was used to measure mouse inflammatory cytokine concentrations in vaginal lavage samples. Inflammatory cytokines included in this study were those shown to be informative in human vaginal microbicide studies [38] and for which there were mouse cytokine reagents available for the Bio-Plex assay. Those tested were: IL-1α, IL-1β, Il-6, KC (murine equivalent of IL-8), MIP-1α, and RANTES. The vagina was lavaged repeatedly (pipetted in and out 20 times) with 50 μl PBS. The lavage fluid was then centrifuged, as described above, to obtain the supernatant which was frozen immediately and stored at -70°C until analyzed. Samples were diluted 1:3 with PBS prior to assay. Cytokine concentrations were determined using the calibration procedure and cytokine reference standards supplied with the Bio-Plex assay. Interference control tests were performed by spiking ten-fold serial dilutions of 2% microbicide samples with defined amounts of cytokine standards. SPS was the only microbicide that inhibited cytokine detection within the concentration range expected to be found in vaginal samples; detection of KC, MIP-1alpha and RANTES was inhibited >50% in the presence of SPS at concentrations ranging from 0.2% to 0.002%.

### Colposcopy

Mice were anesthetized with Avertin (as described above), immobilized with tape on a custom-made platform, and the vagina was held open by means of two small spring-loaded forceps acting like a speculum with four blades. A colposcope (Zeiss OPMI 1-SH) was set at maximum magnification (31×) and focused on the epithelium surrounding and including the cervix. Direct visual observations were made by four observers who were not informed of the agent to which each mouse had been exposed. In addition, photographic images were obtained, and digitized for later analysis. All observations were made with carefully controlled standard illumination, and all images were processed and displayed by identical procedures; no adjustments of color balance or contrast were made to the digitized images.

### Statistics

GraphPad Instat version 3 (San Diego, CA) was used for statistical analysis. Fisher's exact two-sided test was used to analyze numbers of mice infected and uninfected in test groups vs control groups in the protective efficacy and susceptibility experiments. The changes at each time point in the number of cell layers, cellular debris in the lumen, and number of macrophages in the lumen, were compared with those for PBS controls using the unpaired t-test for 2-tailed P value. For cytokines, concentrations in the lavage samples were converted to log_10 _concentrations, and compared with those for PBS controls using the unpaired t-test for 2-tailed P value.

## Results

### Time course of HSV susceptibility after a single application of N9

**Figure 1:**
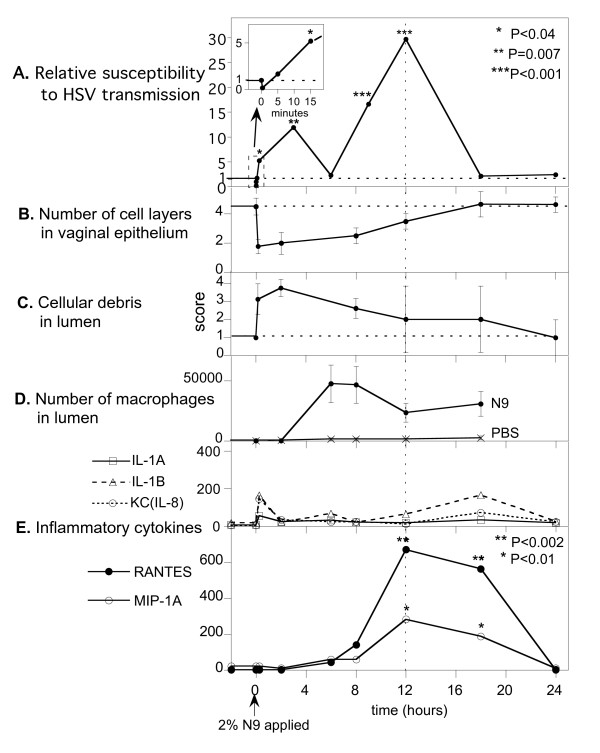
**Vaginal HSV-2 susceptibility and toxic effects vs time after a single dose of 2% N9**. **A. **Relative susceptibility is the effective Infectious Doses the inoculum delivers in N9-exposed mice normalized to the Infectious Doses it delivers in control (PBS) mice. The single dose of N9 produced three successive phases in susceptibility, a brief initial protective phase (reduced susceptibility) followed by two distinct phases of markedly increased susceptibility. *P *values were determined by Fisher's exact two-sided test comparing the numbers of animals infected and uninfected in the test animals vs. the corresponding numbers in control animals treated only with PBS. **B-E. **Error bars indicate standard deviations around the mean. Concentrations of inflammatory cytokines are indicated by geometric means normalized with respect to those for PBS controls. See legend for Fig. 6 for geometric means of PBS controls. **B, C. **Data obtained from histology sections from four locations distributed along the length of the vagina. The score in **C **is a semi-quantitative evaluation of the amount of visible cellular debris in the vaginal lumen. For each point in **B **and **C **at 10 min, 2, and 8 hours, *P *values were <0.001 with respect to PBS control values. (n = 8) **D. ***P *values for all times points from 6 to 18 hours were <0.001 with respect to PBS control values. (n = 20) **D, E. **Data obtained from vaginal lavage samples.

Panel A in Fig. 1 shows susceptibility to HSV as a function of time after delivering a single dose of 2% N9. The small inset shows that immediately after delivery N9 reduced susceptibility about 6-fold (partial protection), but by 5 minutes the mice were no longer protected since the fraction of mice treated with N9 that became infected was larger than the fraction of the control mice treated with PBS. At 15 minutes mice treated with N9 became about 5 times more susceptible than the controls (p = 0.04). Susceptibility rose to a peak at about 3 hours, then declined to the baseline susceptibility of PBS controls at 6 hours. Susceptibility then rose again reaching a major peak at about 12 hours before declining to baseline at 18 and 24 hours. The decrease in susceptibility at 6 hours suggests there are two separate phases of increased susceptibility. Note that at the 12 hour peak, a single dose of N9 increased susceptibility nearly 30-fold (*P *< 0.001), that is, to infect 50% of the N9 exposed mice, a viral inoculum that infects 50% of the control mice would have to be reduced in viral titer by 30-fold.

### Time courses of toxic effects that might correlate with increases in susceptibility

N9 caused rapid exfoliation of epithelial cells, as indicated in Fig 1B by the reduction in number of epithelial cell layers from 4–5 to 1–2 (a loss of about 3 cell layers) and in Fig 1C by the rapid increase in cellular debris in the vaginal lumen. The cell layers slowly regenerated and returned essentially to that of the controls by 18 hours. Note that the initial rapid rise in susceptibility that started at ~5 minutes and peaked at ~3 hours was contemporaneous with the exposure of deeper cell layers in the vagina, and that the subsequent major increase in susceptibility that started after 6 hours, peaked at 12 hours, and ended prior to 18 hours was contemporaneous with the duration in which the layers of cells were regenerating.

N9 also caused a delayed but large influx of macrophages into the vaginal lumen as detected in vaginal lavage samples, Fig 1D. The peak macrophage content of the lumen occurred at about 6–8 hours and somewhat preceded the major peak in HSV susceptibility. Note also that during the initial rapid rise in susceptibility no macrophages were detected in the vaginal lumen, and also that macrophages persisted in the lumen at a greatly elevated level at 18 hours, when the susceptibility had returned to that of the controls.

Fig. 1E shows the time course of inflammatory cytokines recovered in vaginal lavage fluid at the times indicated (each mouse was lavaged once). No Il-6 was detected at any time, and Il-1α did not change detectably at any time. There were non-significant trends towards an immediate increase of IL-1β and KC (murine equivalent to IL-8) followed much later at 18 hours by another non-significant trend toward an increase in IL-1β. In contrast, RANTES and MIP-1α both exhibited a delayed and major increase starting at about 8 hours, peaking at about 12 hours, and persisting at significantly increased levels at 18 hours (a time when the susceptibility had returned to normal).

### Vaginal HSV susceptibility 12 hours after a single application of candidate microbicides

**Figure 2:**
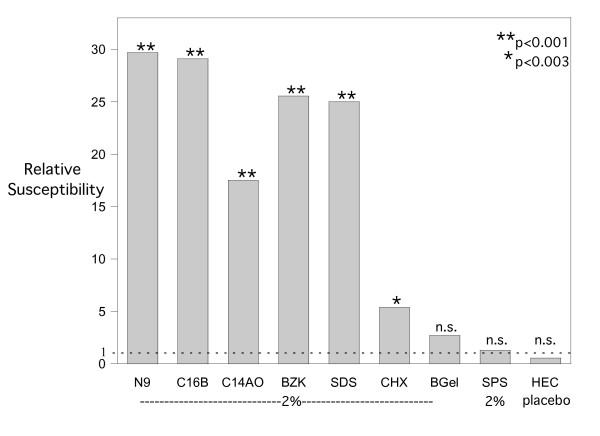
**Vaginal HSV-2 susceptibility 12 hours after a single application of candidate microbicides**. *P *values (from Table 1), determined by Fisher's exact two-sided test as described in the caption for Figure 1. n.s. = not significant.

Since the major peak in susceptibility caused by toxic effects of N9 occurred about 12 hours after delivering this detergent, the other microbicide candidates were also tested 12 hours after applying a single dose. The two detergents in C31G, C_14_AO and C_16_B, were tested separately since they have been reported to differ significantly in their toxic effects [4]. The results are summarized in Fig. 2. All five detergents (N9, C_14_AO, C_16_B, BZK, and SDS) caused comparable major increases in susceptibility (18–29 fold; *P *< 0.001). The susceptibility increases caused by C_14_AO and C_16_B did not differ significantly even though C_16_B has been reported to produce a less intense inflammatory response than C_14_A [4]. The surface-active agent, CHX, caused a smaller but significant 5-fold increase (*P *< 0.003). In contrast, the small susceptibility changes following exposure to BufferGel, SPS, and the HEC placebo were not significant. The data on which Fig 2 is based are listed in Table 1.

**Table 1:** 

agent or PBS	agent then 0.1 ID_50_		PBS then 0.1 ID_50_		PBS then 10 ID_50_		Relative Suscept.	*P*
12 hours earlier	infected	inoculated	infected	inoculated	infected	inoculated		
2% N9	29	42	8	40	35	40	29	<0.001
2% C_16_B	20	30	4	30	25	29	29.1	<0.001
2% C_14_AO	25	41	4	39	34	37	17.5	<0.001
2% BZK	28	40	9	41	37	41	25.5	<0.001
2% SDS	25	37	4	36	34	37	25.0	<0.001
2% CHX	15	45	3	45	35	44	5.4	<0.003
BufferGel	14	52	6	46	39	51	2.7	0.13
2% SPS	5	39	3	34	31	35	1.3	0.7
HEC Placebo	3	32	6	30	30	30	0.5	0.3

2% N9 at time earlier								

5 minutes	8	32	5	32	18	18	1.7	0.5
15 minutes	12	28	4	27	26	28	5.2	0.037
3 hours	26	39	14	41	23	27	12	0.007
6 hours	15	45	10	43	31	39	2.3	0.35
9 hours	22	38	3	37	35	39	16.6	<0.001
18 hours	9	37	5	34	31	36	2.2	0.4
24 hours	8	29	4	29	24	28	2.4	0.3

	2% N9 then 10 ID_50_							

30 seconds	10	18			17	20	0.16	0.07

### A single prior exposure to N9 can abolish its transient protective effect

**Figure 3:**
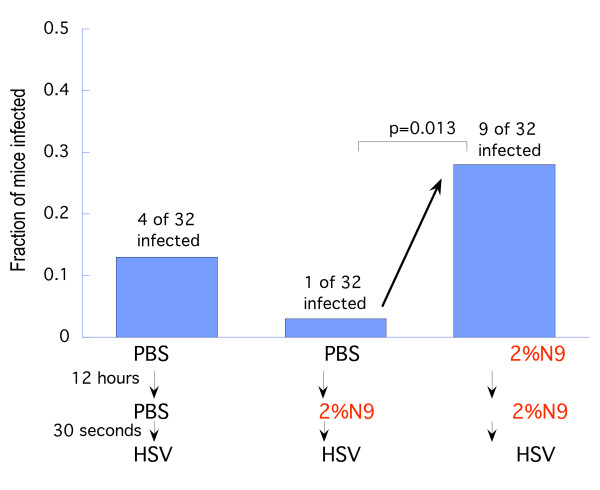
**A single previous exposure to N9 can abolish the brief protective effect of N9**. The low-dose inoculum (0.05 ID_50_) infected only a small fraction of the control group treated twice with PBS (first column), and an even smaller fraction was infected in the control group treated with 2% N9 just before delivering the inoculum (middle column). But a significantly larger fraction became infected in the test group (right-hand column), revealing that the N9 dose delivered 12-hour earlier abolished the protective effect of the N9 dose delivered just before the inoculum: *P *= 0.013 by Fisher's exact two-sided test.

The disappointing results of the clinical trials of N9 products for HIV prevention led to the suggestion that persistent toxic effects might increase susceptibility during coital events when the product was not used. If this is the major problem caused by N9 toxicity, then "user failure" is of crucial importance. However, the present results suggest an additional possibility, namely that detergent toxicity may cause such rapid and large increases of susceptibility that even when used correctly they may fail to protect. Most N9 products are recommended to be used within 1 hour of application, but as shown in Fig. 1A, N9 significantly increased susceptibility within 15 minutes of application. A second test of the hypothesis that detergents may not protect even when used correctly is shown in Fig. 3.

Mice were exposed to a single dose of 2% N9, or PBS, and then 12 hours later were treated with N9, or PBS, just before delivering a *low-dose *viral inoculum (0.05 ID_50_). As can be seen in Fig. 3, the low-dose inoculum infected 4 of 32 control mice (exposed twice to PBS), and 1 of 32 positive control mice (PBS followed 12 hours later by 2% N9 delivered just prior to the inoculum). In contrast, 9 of 32 test mice previously exposed to N9 became infected, even though in this test group a second dose of N9 was delivered just prior to the low-dose inoculum to mimic correct use of a microbicide: (9 of 32 vs 1 of 32; P = 0.013). Thus a single earlier dose of N9 abolished the transient protective effect of the subsequent dose of N9 even when used correctly.

### Acute toxic effects of detergents

**Figure 4:**
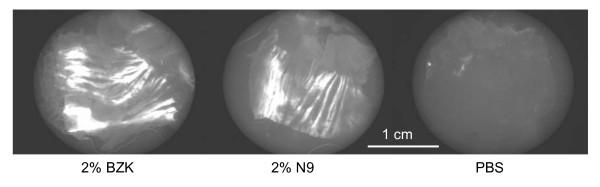
**Surface distribution of acute toxicity to vaginal epithelium caused by a 10-minute exposure to detergents**. Fluorescent intensity of the 'dead cell' dye YOYO-1 is proportional to the density of cell nuclei in the epithelium whose membranes were disrupted by the detergents. The brightly stained swaths of epithelium are regions in which virtually every cell nuclei became stained with YOYO-1. The darker regions became visible as the vagina was gently stretched and flattened to unfold the rugae.

Detergents can rapidly disrupt the membranes of living cells exposed on the epithelial surface, and Fig. 4 shows the effects of a 10-minute exposure to 2% BZK, 2% N9, and PBS applied to the vagina of progestin-treated mice. The entire vaginas, opened and flat-mounted, were stained with the "dead cell" dye YOYO-1 (Molecular Probes, Eugene, OR) that makes nuclei of cells with disrupted membranes intensely fluorescent. Both detergents caused large swaths of the epithelial surface to become intensely fluorescent, regions in which virtually every cell nucleus became highly fluorescent. In contrast, PBS caused little detectable damage.

The images graphically reveal the extent of acute damage caused by these detergents to the living cells on the epithelial surface, unprotected by layers of senescent squamous cells. Note that the swaths of fluorescent cell nuclei visible in this figure were in cells located in *deeper *layers of the epithelium since the detergents caused the outermost cell layers to be rapidly shed, as shown above in Fig. 1B and 1C. Also noteworthy is that the detergents failed to enter several regions of the vaginal surface, as indicated by the dark, unstained bands. These bands were located on the interior surfaces of the vaginal rugae (folds); these bands became fully visible only when the vagina was flattened (or stretched laterally) sufficiently to spread out the rugae. Thus the 10-minute exposure to the detergents was not sufficient for detergents to enter into the rugae of the vagina. This result was similar to an earlier study using ethidium homodimer-1 instead of YOYO-1 [23]. In subsequent studies (data not shown), we found that even if a large ball is placed on the tip of the pipet to help spread out the rugae, and the pipet is stirred with a variety of motions, the detergents still fail to enter many of the rugae. This incomplete "deployment" of vaginal microbicides into the many folds of the vaginal epithelium probably contributes to the inability to achieve complete protection in tests of candidate microbicides in animal models [23]. Moreover, as shown in the following section, incomplete deployment probably contributes to incomplete protection even when microbicide agents are delivered at concentrations much higher than needed to inactivate pathogens in vitro.

### Selectivity index in vivo

Animal models provide the best available method for predicting whether a candidate microbicide will be sufficiently selective to merit tests in human clinical trials. An *in vivo *selectivity index can indicate whether or not a test agent can provide protection without causing unacceptable toxic effects.

**Figure 5:**
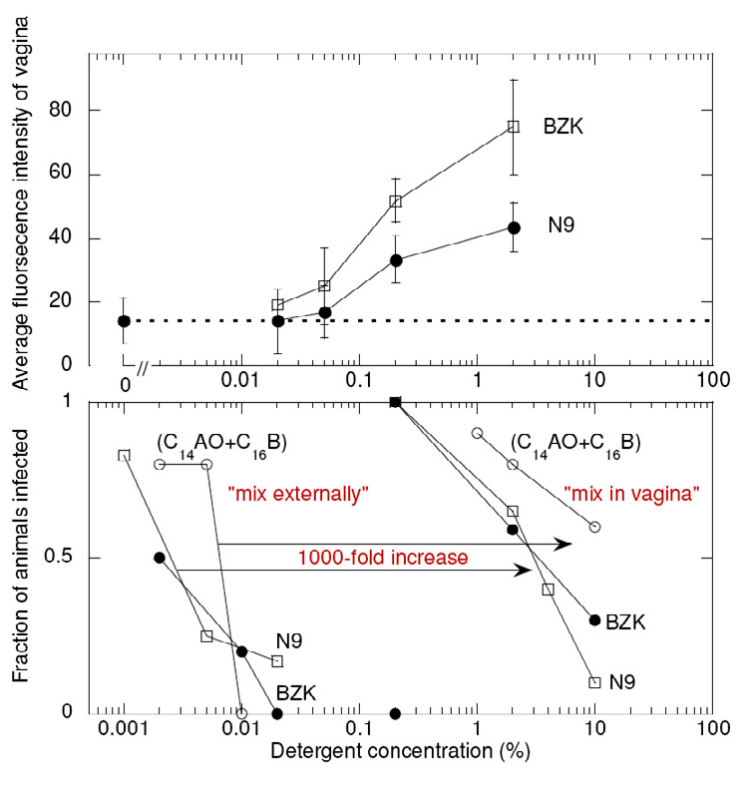
**Acute toxicity, and fraction of mice infected, vs detergent concentration**. The upper panel shows the average fluorescent intensity of the YOYO-1 stained vagina as a function of the concentration of detergent to which each vagina was exposed for 10 minutes. The lower panel shows the fraction of mice infected using two different test methods: 'mix externally' fully exposes HSV in a high-dose inoculum (10 ID_50_) to the microbicide prior to inoculation, and 'mix in vagina' mimics human use of a microbicide, the microbicide is delivered to the vagina before delivering the high-dose (10 ID_50_) inoculum. The two detergents in C31G, (C_14_AO+C_16_B), were combined at equal w/v concentrations and plotted at the total detergent concentrations shown.

Fig. 5 illustrates methods for determining a selectivity index. The upper panel shows a measure of acute toxicity, the average fluorescence intensity of the entire vagina stained with YOYO-1 as above (Fig. 4) as a function of the concentration of the detergent to which the vaginas had been exposed for 10 minutes. BZK and N9 caused detectable increases in fluorescence starting at about 0.05% and markedly increased fluorescence at 0.2% and above, indicating major regions of epithelial cells had disrupted membranes. (Other detergents we tested, especially SDS, an anionic detergent, significantly blocked fluorescence by YOYO-1, a cationic dye, and hence acute toxicity caused by these detergents could not be evaluated with this method. Apparently, despite the extensive and repeated washing procedure, significant amounts of detergents must have been retained by epithelial cells, perhaps by being intercalated into cell membranes at concentrations too low to solubilize the membranes but high enough to interfere with fluorescence of YOYO-1.)

The lower panel in Fig. 5 shows two methods for testing the protective efficacy of a candidate microbicide in the mouse HSV model. The "mix externally" method is similar to cell-based *in vitro *tests; the viral inoculum is thoroughly mixed together with the test agent *before *the mixture is delivered to the vagina. This insures that the viral inoculum is well exposed to the test agent before the virions can contact target cells. The "mix in vagina" method is designed to mimic human use of microbicides; the candidate microbicide is delivered to the vagina first, and then the viral inoculum is delivered.

As expected on the basis of cell-based tests, the detergents were significantly protective in the "mix externally" method at the minimal concentrations that inactivate HSV and HIV virions *in vitro*, ~0.03% [31,33]. At a slightly higher concentration, ~0.05%, the detergents disrupted cell membranes in the epithelium, as indicated by increased YOYO-1 fluorescence of the vagina. In marked contrast, but also as expected from previous tests in animal models [26,39,40], in the "mix in vagina" method the detergents failed to protect unless delivered at concentrations ~1000-fold higher (~3%). By comparing the upper and lower panels in Fig. 5 it can be seen that detergents became acutely toxic at much lower concentrations than the concentrations needed for significant protection. Most important, the detergents provided only partial, if any, protection at 2%, a concentration at which all the detergents markedly increased HSV susceptibility as shown above in Fig. 2. Thus in this mouse HSV model there was no concentration at which N9, BZK, C_14_AO, C_16_B, and SDS were protective without causing unacceptable toxicity.

### Inflammatory cytokines

**Figure 6:**
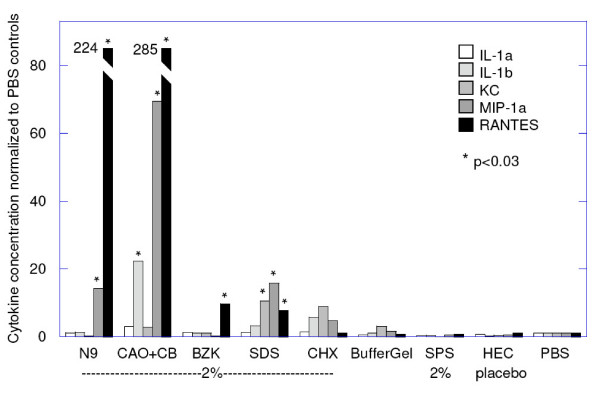
**Inflammatory cytokines 12 hours after a single exposure to candidate microbicides**. Geometric means for each agent are shown normalized by the results in PBS controls as follows, in ng/ml of lavage fluid: IL-1α, 16; IL-1β, 50; KC, 42; MIP-1α, 20; RANTES, 3. Thus N9 increased the concentration of RANTES 224-fold, from 3 to 672 ng/ml in lavage fluid. (The lavage procedure is estimated to have diluted vaginal secretions by 10–50-fold.) (CAO+CB) = 1%CAO+1%CB = 2% detergent.

Prior to Phase II/III efficacy trials, there is a critical need for tests that can be performed in Phase I safety trials to screen out candidates likely to cause unacceptable toxicity. One such screening test is to observe the release of inflammatory cytokines since inflammatory responses are likely to increase susceptibility to HIV and other pathogens [10,38]. In the mouse HSV model, toxic effects that increase susceptibility can be directly measured and correlated with releases of inflammatory cytokine responses to help determine which increases in cytokines correlate best with increases in susceptibility to HSV infections. Therefore, in experiments performed in parallel with those shown in Fig. 2, vaginal lavage samples were obtained 12 hours after a single exposure to the candidate microbicides and analyzed for inflammatory cytokines. The results are summarized in Fig 6. The detergents were all tested at a total concentration of 2%, since this was the minimal concentration at which these detergents provided partial transient protection. Twelve hours after a single application, all detergents tested produced a significant increase in one or more inflammatory cytokines: All significantly increased RANTES; N9, (C_14_AO+C_16_B), and SDS significantly increased MIP-1α. In addition, (C_14_AO+C_16_B) significantly increased IL-1β, and SDS significantly increased KC (murine equivalent of Il-8). Thus all detergents, as tested here, caused significant increases both in HSV susceptibility and release of one or more inflammatory cytokines. Chlorhexidine (CHX) caused a less marked increase in HSV susceptibility and only statistically insignificant trends in Il-1β, KC, and MIP-1α. In contrast to the detergents, BufferGel, SPS, and the HEC placebo did not significantly increase susceptibility nor cause a significant release of inflammatory cytokines. SPS also did not increase susceptibility, but cytokine proinflammatory results are inconclusive because unlike the other agents, SPS significantly interfered with the detection of several of the cytokines in the Bioplex assay.

In summary, by observing a broad range of inflammatory cytokines there emerged a significant, but non-quantitative correlation between susceptibility-increasing toxicity and the elevation of inflammatory cytokines. But note that the time-courses were *not *well correlated.

### Colposcopy fails to detect N9 toxicity that increases susceptibility

**Figure 7:**
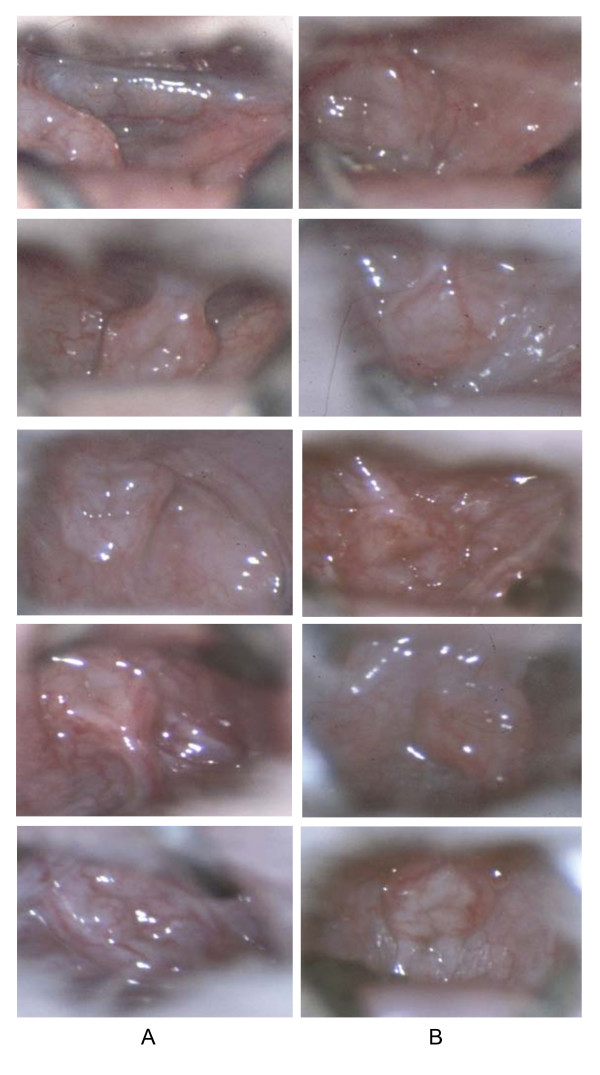
**Colposcopic images of mouse vaginas 12 hours after a single exposure to 2% N9, or PBS**. The colposcope (Zeiss OPMI 1-SH) was set at maximum magnification (31×) and focused on the vaginal area surrounding the cervix. Column A, 2% N9, column B, PBS controls.

Any candidate microbicide that causes colposcopically detectable vaginal toxicity is likely to be screened out prior to Phase II/III efficacy trials. But it is not known whether colposcopy can detect all toxic effects that greatly increase susceptibility to HSV or HIV. Fig. 7 shows high-magnification colposcopic images obtained from 10 mice treated either with a single application of 2% N9 or PBS as a control. These images were obtained 12 hours after delivering the detergent (or PBS), the time at which the susceptibility-increasing toxicity of N9 reaches its peak. Four observers independently attempted to use colposcopy to distinguish mice treated with N9 from those treated with PBS. Attention was focused on detecting erythema, ulcerations, and widened capillaries with more diffuse borders. Neither by direct visual examination with the colposcope, nor by careful examination of digitized, greatly enlarged photographic images, could the observers do better than chance in distinguishing the N9-exposed mice from the control mice.

## Discussion

### Effects of N9 in mice and humans

N9 was thoroughly studied here since it is the microbicidal detergent for which there is the most clinical evidence regarding both its contraceptive efficacy against sperm [41] and also its failure to provide protection against STD pathogens some of which, like HIV, are potently and rapidly inactivated by this detergent [42,43]. N9 is highly potent *in vitro *against both sperm [34] and HIV [33](~0.02% inactivates both in seconds), and N9 is an effective contraceptive. This has led to the conjecture that despite its toxic effects N9 might protect against HIV *if used correctly every time*. However, the results in this mouse model indicate that even with *correct *use, N9 may fail to protect against vaginal HSV infections. For most N9 products it is recommend that coitus take place within 1 hour of application, but as shown in Fig. 1, the duration of protection in the mouse lasted only ~5 minutes, and susceptibility increased significantly by 15 minutes, even though most of the detergent was still present (see [44]). This suggests that in human use susceptibility might actually increase soon after the first application of the detergent. Moreover, with repeated use, the prolonged susceptibility-increasing toxic effects of N9 might abolish even the brief duration of protection it might otherwise provide, as shown in Fig 3.

Taken together with the rectal studies by Phillips and colleagues [20] the increases in vaginal susceptibility caused by all five detergents and by CHX cast doubt on the suitability of detergents and surface-active agents for vaginal as well as rectal protection against HSV. Single applications of N9 and K-Y^® ^jelly to the mouse uterus cause long-lasting toxic effects (exfoliation and regeneration still evident after 48 hours) [9]. CHX, the preservative in K-Y^® ^jelly, not only increases vaginal susceptibility to HSV but also causes a major, and long-lasting (3 days) increase in susceptibility to chlamydia [23]. Since vaginal applications of the detergents cause significant release of inflammatory cytokines in mice, rabbits [10] and women [38], these detergents may also increase susceptibility to HIV infections (see [10,38]). In contrast, both non-detergent candidates tested here, SPS and BufferGel, when applied at a protective dose [26,29], did not cause significant toxic effects.

### Epithelial exfoliation and regeneration correlates best with HSV susceptibility

We attempted to identify observable toxic effects that could be used in preclinical and clinical Phase I safety trials to screen out candidate microbicides that cause susceptibility-increasing toxic effects. These included observations of epithelial exfoliation (desquamation) and regeneration, vaginal entry by macrophages, release of inflammatory cytokines, and colposcopy. The toxic effect that best correlated with the time-course and duration of increased HSV susceptibility was the time course of the changes in thickness of the epithelium. The initial rapid increase in susceptibility occurred during the initial rapid exfoliation of epithelial cells (thereby exposing cells deeper in the epithelium). This result is similar to the rapid exfoliation caused by N9 applied to the columnar epithelium of the mouse uterus [9] and mouse rectum [20]. N9 rapidly increases rectal susceptibility in the mouse. Based on the dose-response results reported by Phillips and Zacharopoulos [20], the lowest dose viral inoculum they used, 5 × 10^3 ^PFU, delivered ~0.05 ID_50 _to the controls, but acted effectively as ~5 ID_50 _when delivered 5 minutes after applying 2 % N9 to the rectum. Thus 2% N9 increased rectal HSV susceptibility by ~100 fold within 5 minutes of application. The columnar epithelium of the mouse rectum appears to regenerate within ~1 hour [20]. In contrast, the columnar epithelium of the mouse uterus regenerates in ~72 hours [9]. Since the vaginal epithelium became maximally susceptible at ~12 hours, when its cell layers were regenerating, our results suggest that rectal and uterine susceptibility might become maximal during the markedly different rates at which their epithelial cell layers regenerate.

### Nectin-1

A possible mechanism for both the early and late phases of increased susceptibility that occur following epithelial exfoliation caused by detergents is suggested by the following findings regarding nectin-1, a major cell-surface entry receptor for HSV: 1) Mice are susceptible to HSV only during the stages of the estrous cycle when the vaginal epithelium expresses nectin-1, and the most susceptible stage can be maintained by treatment with Depo-Provera [45], 2) Incubating HSV with nectin-1 prior to vaginal inoculation blocked infection [45], 3) In the intact epithelium nectin-1 is primarily localized in the lateral surfaces between adjacent epithelial cells, a location where it is relatively inaccessible to HSV. But upon epithelial disruption by low calcium, nectin-1 migrates from the lateral surfaces and becomes accessible to HSV on the apical surface, increasing viral entry by HSV [46]. In the human vagina, nectin-1 is expressed most intensely in deeper cell layers, the stratum spinosum, the layer of maturing cells just above the basal cells, and is expressed closer to the luminal surface during epithelial regeneration in the secretory phase of the cycle [45]. This suggests that rapid desquamation will rapidly increase access of HSV to cells whose surfaces have more nectin-1, and also that during regeneration there will be more nectin-l on the epithelial surface. Nectin-1 is also heavily expressed on cell surfaces during epithelial remodelling in the mouse embryo [46], which again suggests nectin-1 may be heavily expressed during the regeneration of vaginal epithelial cell layers.

Thus nectin-1 provides a possible mechanism to explain both the rapid initial increase in susceptibility and for the later major increase in susceptibility (compare the time-course for susceptibility, Fig. 1A, with the number of cell layers, Fig 1B.). The rapid initial increase in susceptibility was contemporaneous with the exposure of deeper layers in the epithelium, and the later prolonged increase in susceptibility occurred contemporaneously with epithelial regeneration.

Taken together, these results suggest that women may become more susceptible to HSV within minutes of applying a microbicide that causes rapid exfoliation of columnar epithelium and may become even more susceptible throughout the duration of epithelial regeneration. Human columnar epithelial cells are target cells for HSV, and in the absence of trauma or open sores or ulcers, the most susceptible sites are likely to be the columnar epithelial cells in regions of cervical ectopy and in the endocervix. Uterine peristalsis transports vaginal fluids, including sperm [see [47]] and microbicide gels [48-51], to the columnar epithelium of upper tract. Thus microbicides (and pathogens) both contact columnar epithelium of the upper tract. Even though the vaginal epithelium is protected by multiple layers of dead and dying squamous cells, toxic effects of microbicides applied to columnar epithelium should be carefully considered in the design of Phase I trials.

### Which toxic effects related to HSV susceptibility might usefully be monitored in Phase I trials of candidate microbicides?

#### Exfoliation

Monitoring changes in the thickness of the columnar epithelium in women would require biopsy specimens of ecto- or endocervical tissue taken shortly before and after exposure to the test agent. However, optical coherence tomography (OCT, [52]) might provide a non-invasive method for detecting ecto-cervical exfoliation, and it might also be possible to detect rapid epithelial exfoliation by collecting lavage or swab samples directly from the ectocervix and cervical os before and shortly after applying the candidate microbicide and looking microscopically for epithelial sheets.

#### Leukocyte entry

Fichorova, Tucker, and Anderson [38] reported that N9 caused macrophages and neutrophils to enter the human vaginal lumen in large numbers. Milligan et al, [8] reported that both N9 and SDS caused the same to happen in the mouse vagina, and we confirmed their findings for N9 and macrophages in this study. Milligan et al. also reported that two non-detergent candidate microbicides based on sulfated and sulfonated polymers, T-PSS and PRO 2000, did not cause significant increases in vaginal leukocytes even after repeated doses. Thus leukocyte entry into the lumen is a toxic effect that correlates in this mouse model with increased HSV susceptibility. However, unlike changes in epithelial thickness, the time-course of leukocyte entry does not correlate well with the increases in susceptibility: During the initial increase in susceptibility there was no significant increase in the numbers of leukocytes in the vagina, and at 6 hours, when the number of macrophages had markedly increased, HSV susceptibility returned essentially to normal. At 18 hours, HSV susceptibility again returned to normal but high numbers of both macrophages and neutrophils persist for many more hours [8].

#### Colposcopy

High-magnification colposcopy surprisingly failed to distinguish between mice exposed 12 hours earlier to N9 from controls treated with PBS. This result does not argue against performing colposcopic exams, but suggests instead that this method may not be adequate to detect certain toxic effects that cause major increases in susceptibility to HSV.

#### Inflammatory cytokines

Inflammatory cytokines are now being investigated during Phase I trials of vaginal microbicide candidates, not only as sensitive markers for epithelial damage, but also because they provide insight into important biological changes that may occur in the vagina as a result of microbicide administration. For example, increased chemokine concentrations correlate with an influx of immune and inflammatory cells into the vaginal mucosa. These cells can play a role in host immune defence, but CD4+ immune cells are also target cells for the HIV-1 virus, and their increased numbers could promote HIV-1 infection. Likewise, increased concentrations of Il-1 can activate NF-KB, an intracellular transcription signal that leads to the production of other inflammatory cytokines, and can promote HIV-1 shedding through activation of the HIV-1 LTR.

The results of the present study lend further support for screening candidate microbicides to insure they do not cause significant increases in inflammatory cytokines. The results in mice suggest that several different cytokines, and durations of exposure, should be investigated since no single cytokine served as a reliable predictor of significant toxicity at the time points chosen for this study. For example, although the detergents all caused a significant increase in RANTES, they did so to markedly different degrees at the 12-hour time point (N9 increased RANTES ~30-fold more than did SDS). Moreover, the major differences in cytokines released, as shown in Fig. 6, do not correlate with the almost *uniform *increase in susceptibility caused by all five detergents as shown in Fig. 3. Note also that in the mouse, even though a single dose of N9 markedly increased susceptibility to HSV it did not cause a detectable release of Il-6 and only insignificant trends occurred in Il-1α, Il-1β, and KC(Il-8), further emphasizing the lack of obvious correlation between cytokine release and HSV susceptibility. In the rabbit toxicity model Fichorova et al [10] found that N9, BZK, and SDS caused marked, and markedly different, increases in Il-6, IL-1β, and IL-8 when observed 24 hours after the first, second, and third daily application. In humans [38] a single application of N9 did not cause a detectable increase in any of the soluble mediators observed in lavage samples obtained after 12, 36, and 60 hours: IL-1α, IL-1β, IL-8, IL-1_ra_, TNF-RI, TNF-RII (but at 12 hours, there was a significant decrease in SLPI). However, after 3 daily exposures, several mediators increased significantly: IL-1α and IL-1β (at 12, 36, and 60 hours after the last application), IL-8 (at 36 hours), MIP-1β (at 60 hours), TNF-RII (at 36 hours), neutrophil elastase (at 12 and 36 hours), and SLPI significantly decreased (at 12, 36 and 60 hours).

Human columnar endocervical epithelial cells are more sensitive to toxic effects of N-9 in vitro and release more proinflammatory cytokines when damaged than do vaginal epithelial cells[10]. Similarly, in Swiss-Webster mice, the cervical columnar epithelium is more sensitive to the toxic effects of detergents than stratified squamous vaginal epithelium [5,6]. A major difference between vaginal lavage samples obtained from humans and the progestin-treated mice in the present study is the relatively small surface of columnar epithelium in humans that contributes to the lavage sample. Thus lavage samples from progestin-treated mice in which the entire vaginal epithelium is covered with living cells may provide a more sensitive measure of the effects of candidate microbicides on epithelia that are not protected by layers of squamous cells. This may explain why the increases in cytokines caused be single applications of the detergents were readily detectable in the mouse but not in humans.

Finally, our study also highlights the need to test microbicide agents and formulations for inhibitory effects on cytokine detection. SPS may have produced false negative readings in the cytokine test because it had a strong inhibitory effect on cytokine detection in the Bioplex assy.

### Limitations of this study

Even though the mechanism of infection and pathogenesis of genital HSV is very similar in both humans and mice, there is still uncertainty predicting results in humans based on results in mice.

Pre-treatment with progestin (Depo-Provera) markedly increases HSV-2 susceptibility in mice, but it has not been documented whether it has this effect in humans. However, epithelia in both species that are not protected by a squamous layer are likely to be more susceptible to HSV.

Effects of formulation were not examined in this study, and different formulations may have different effects on toxicity (and efficacy).

Toxic effects of repeated exposures were not examined in this study. It is apparent that a single exposure to detergents and CHX caused significant toxicity, but other candidate microbicides may not cause significant toxicity unless applied repeatedly.

Results in this mouse/HSV model may not predict the effect of microbicides on human transmission of HIV, since there are significant differences in the mechanisms of transmission. In view of the 20–30 fold increase in HSV susceptibility a single dose of detergent causes in the mouse vagina, and the >100 fold increase in susceptibility N9 causes in the mouse rectum [20], it may be advisable to develop a primate model that directly detects whether a candidate microbicide increases susceptibility to SIV or SHIV since the surrogates now being monitored in animal models and Phase I trials may not reveal serious alterations in susceptibility.

## Conclusion

1. The mouse model for vaginal transmission of HSV-2 can be used to detect toxicities caused by candidate microbicides that increase susceptibility to infection.

2. Detergents applied at a minimally protective dose caused rapid, prolonged, and major increases in vaginal HSV-2 susceptibility.

3. SPS and BufferGel are examples of candidate microbicides that protect against HSV infections without causing susceptibility-increasing toxic effects in this model.

4. In screening for toxic effects of microbicides in Phase I safety trials, it may be important to monitor a broad range of inflammatory cytokines vs time, and exfoliation of columnar epithelium.

## Competing interests

RAC and TRM hold equity in ReProtect, Inc., which is developing BufferGel as a spermicidal microbicide. Authors declare there are no other competing interests.

## Authors' contributions

RAC and TRM initiated the study and participated in the design and analysis throughout. RAC drafted the manuscript, and all authors read, helped revise, and approved the final manuscript. TH helped develop the procedures used in the mouse HSV model, and performed most of the experiments using this model. XXW performed some of the susceptibility tests that determined the time-course of susceptibility, and designed and performed the histology, macrophage entry, and colposcopy experiments, RA helped develop and perform the acute toxicity "dead cell" assay, and the inflammatory cytokine assays were performed in DJA's laboratory.

## Pre-publication history

The pre-publication history for this paper can be accessed here:


